# First Report of Laparoscopic Radical Nephroureterectomy Following Extraperitoneal Tubeless Umbilical Cutaneous Ureterostomy

**DOI:** 10.1002/iju5.70171

**Published:** 2026-03-19

**Authors:** Tsubasa Kondo, Toshifumi Tsurusaki, Akihiro Asai, Lisa Komuro, Kuniko Abe

**Affiliations:** ^1^ Department of Urology Japanese Red Cross Nagasaki Genbaku Hospital Nagasaki Japan; ^2^ Department of Pathology Japanese Red Cross Nagasaki Genbaku Hospital Nagasaki Japan

**Keywords:** cutaneous ureterostomy, cystectomy, urinary diversion, urothelial carcinoma

## Abstract

**Introduction:**

Extraperitoneal tubeless umbilical cutaneous ureterostomy is a useful urinary diversion technique for patients with shortened ureters or high risk of upper urinary tract recurrence. However, radical nephroureterectomy following this diversion has not been previously reported.

**Case Presentation:**

A 67‐year‐old man underwent radical cystoprostatourethrectomy with extraperitoneal tubeless umbilical cutaneous ureterostomy for high‐risk urothelial carcinoma with bilateral ureteral shortening. Twenty‐four months later, left‐sided upper tract urothelial carcinoma was diagnosed, and laparoscopic left radical nephroureterectomy was performed. Although dense adhesions necessitated partial small‐bowel resection, complete nephroureterectomy was achieved without major complications.

**Conclusion:**

To the best of our knowledge, this is the first report of radical nephroureterectomy performed after umbilical cutaneous ureterostomy. This urinary diversion may represent a reasonable option for selected patients at high risk of upper urinary tract recurrence.

## Keynote Message

Extraperitoneal tubeless umbilical cutaneous ureterostomy may be a reasonable urinary diversion option for patients with shortened ureters or at high risk of upper urinary tract recurrence. We report the first case of laparoscopic radical nephroureterectomy performed after this procedure. Despite requiring partial small‐bowel resection, complete ureteral removal was achieved, suggesting that this approach may facilitate subsequent upper urinary tract surgery in selected high‐risk patients.

**Abbreviations**
BCG: Bacillus Calmette‐Guerin
CIS: carcinoma in situ
CU: cutaneous ureterostomy
RC: radical cystectomy
RNU: radical nephroureterectomy
UC: urothelial carcinoma
UTUC: upper tract urothelial carcinoma

## 1 Introduction

**FIGURE 1:**
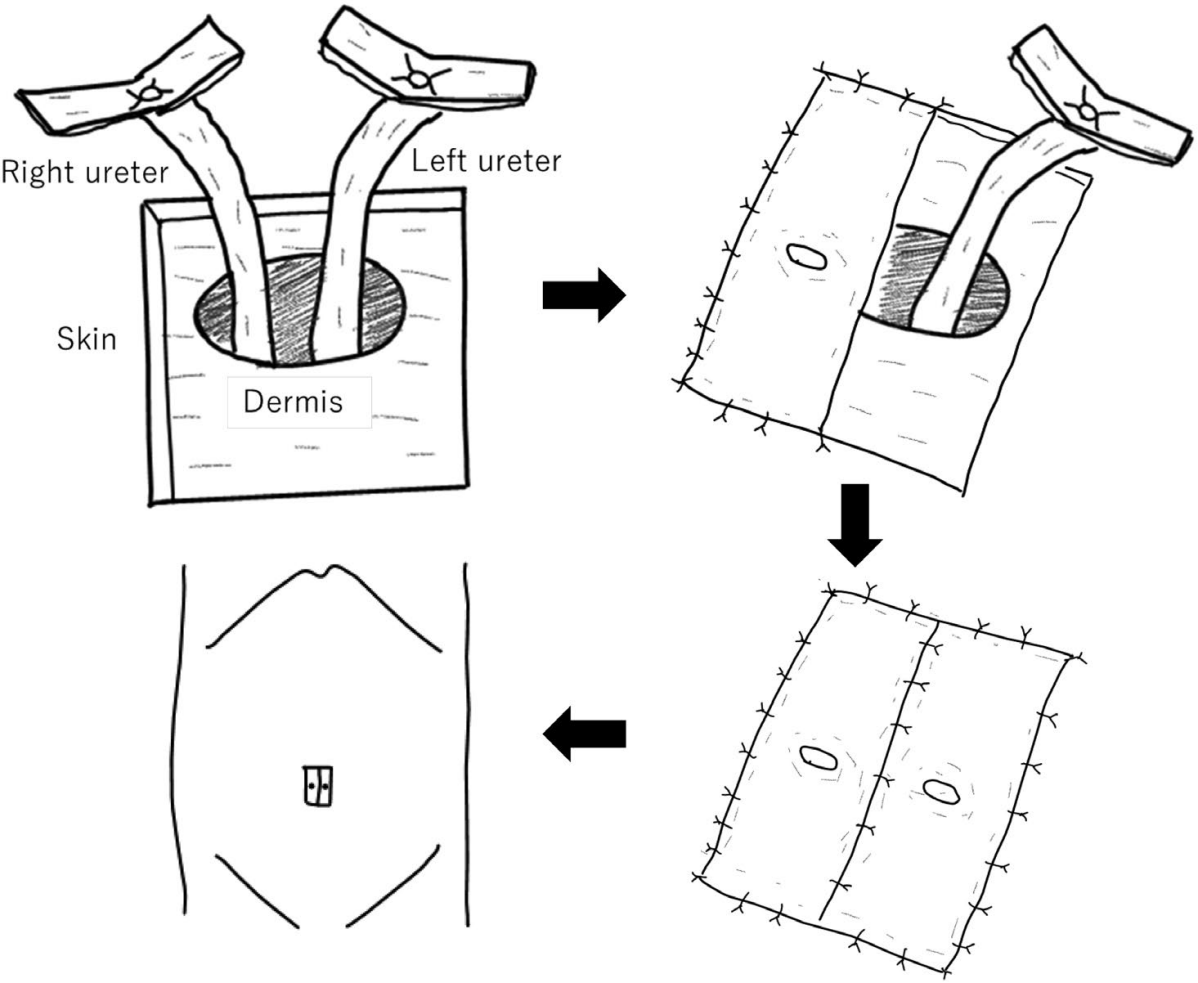
Schematic illustration of extraperitoneal bilateral tubeless umbilical cutaneous ureterostomy using Toyoda method. Both ureters are brought ventrally through an extraperitoneal route and matured into a single umbilical stoma without ureteroureteral or ureteroileal anastomosis.

The risk of upper urinary tract recurrence after radical cystectomy (RC) is significantly higher in patients with carcinoma in situ (CIS) or positive ureteral margins [1]. Surgical management of upper tract urothelial carcinoma (UTUC) contralateral to the stoma is technically challenging and may increase the risk of insufficient distal ureteral resection or positive surgical margins. This challenge arises because, in patients with an ileal conduit, orthotopic neobladder, or cutaneous ureterostomy (CU), one ureter typically courses dorsally to the sigmoid colon and diverts toward the contralateral stoma. To address this issue in patients with shortened ureters, we previously developed a novel urinary diversion technique involving extraperitoneal tubeless umbilical CU (Figure 1) [2]. To our knowledge, this is the first report of radical nephroureterectomy (RNU) performed after this urinary diversion, suggesting its potential clinical utility.

## 2 Case Presentation

**FIGURE 2:**
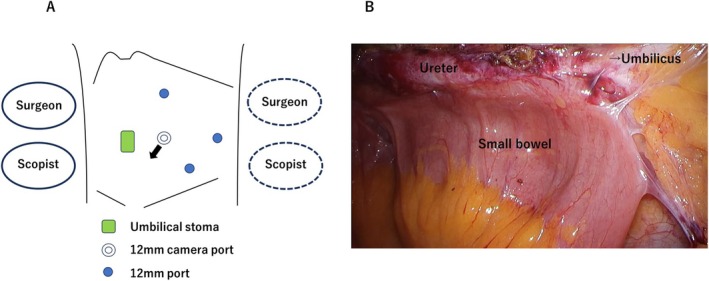
(A) Port placement and positions of the surgeon and scopist during laparoscopic left radical nephroureterectomy. The black arrow indicates the direction of the laparoscopic view shown in panel B. The dashed outlines represent the positions of the surgeon and scopist after repositioning to the patient's dorsal side during ureteral dissection toward the umbilical stoma. (B) Laparoscopic image obtained during left radical nephroureterectomy from the viewing direction indicated in panel A. Dense adhesions between the small bowel and distal ureter are observed.

**FIGURE 3:**
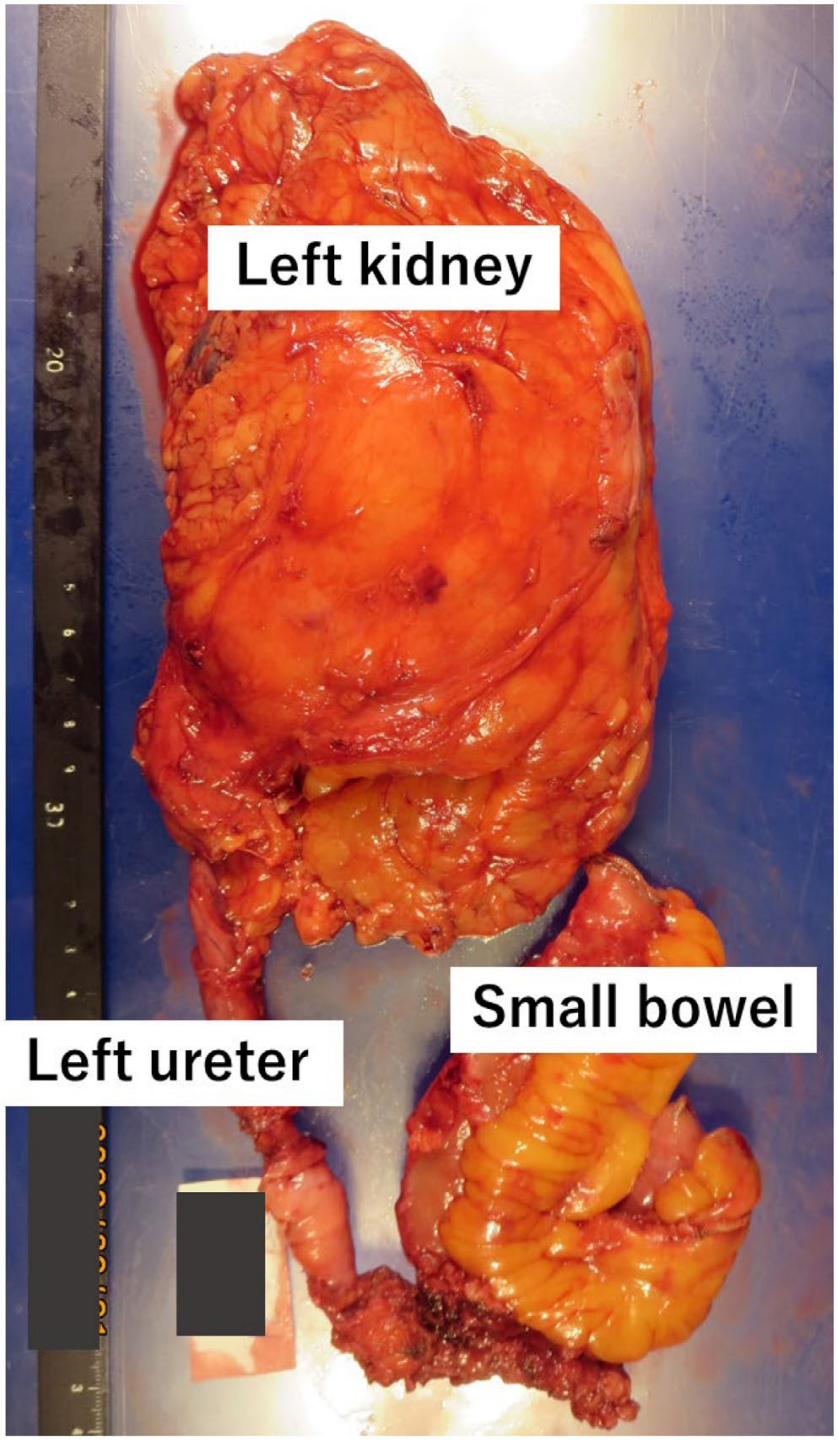
Macroscopic appearance of the specimens obtained from left radical nephroureterectomy and small bowel following combined resection.

A 67‐year‐old man was diagnosed with high‐grade pT1 urothelial carcinoma (UC) with concomitant CIS of the bladder and prostatic urethra following transurethral resection. The primary tumor involved the bladder neck and right lateral wall, and the right ureteral orifice was not identifiable. After discussing treatment options, the patient opted for RC. Despite the absence of preoperative hydronephrosis, a positive distal right ureteral margin was anticipated, and ileal conduit diversion was initially planned. Intraoperatively, both distal ureters were ligated and transected, and frozen‐section analysis revealed urothelial dysplasia in the right ureter and CIS in the left ureter. Based on these findings, an additional 2 cm of each ureter was resected proximally. However, a second frozen‐section analysis yielded similar results. Given these findings, we deliberated whether to proceed with left RNU and how best to manage urinary diversion. Ultimately, we elected to preserve both kidneys and planned postoperative bilateral upper urinary tract Bacillus Calmette–Guérin (BCG) instillation therapy. Repeated frozen‐section analyses resulted in ureteral shortening, and the left ureter could not reach the planned right‐sided stoma. Therefore, an umbilical CU was constructed to accommodate the shortened ureter and facilitate future access to the upper urinary tract. An additional 1.5 cm of the left ureter was resected because of a positive margin. The final pathology confirmed high‐grade CIS in the bladder and prostatic urethra with negative surgical margins, including both ureters. The patient had no postoperative complications and declined postoperative BCG instillation therapy. Postoperative surveillance consisted of urinary cytology and ultrasonography every 2 months, with periodic computed tomography. Although cytology was initially negative, atypical cells appeared at 6 months, followed by progressive left hydronephrosis. At 23 months postoperatively, selective left‐sided cytology was positive. Despite the absence of an obvious filling defect on antegrade pyelography, these findings supported a diagnosis of left‐sided UTUC, and laparoscopic RNU was performed at 24 months. A transperitoneal approach was selected because retroperitoneal dissection was considered difficult due to the anticipated adhesions. The procedure was performed in the same lateral decubitus position routinely used for laparoscopic left nephrectomy. After renal vessel control and kidney mobilization, the ureter was dissected. The surgeon and scopist were initially positioned ventrally but repositioned dorsally during dissection toward the umbilicus (Figure 2A). A ureteral catheter was placed in the right ureter to prevent injury to the contralateral CU. Dense adhesions between the small bowel and distal ureter were observed (Figure 2B). Because safe dissection was not feasible and tumor invasion could not be excluded, en bloc partial small‐bowel resection with end‐to‐end anastomosis was performed (Figure 3). The left umbilical stoma was excised, and the kidney and ureter were completely removed. The operative time was 4 h 47 min with an estimated blood loss of 20 mL. Pathological examination demonstrated widespread high‐grade CIS of the renal pelvis and ureter without extramural extension or small‐bowel invasion. Given the absence of direct tumor infiltration and the presence of fibrous adhesions between the ureter and small bowel, the adhesions were considered inflammatory and likely mediated via the peritoneum. The patient's postoperative course was uneventful, and no recurrence was observed 13 months after RNU.

## 3 Discussion

Urinary diversion following RC is usually performed using an ileal conduit or orthotopic neobladder. However, in cases where the intestine cannot be used because of prior abdominal radiation therapy, intestinal adhesions, or poor general condition, a less invasive CU that does not require intestinal resection may be selected. CU is generally technically simpler and requires a shorter operative time than intestinal urinary diversion procedures [3]. A stoma is usually created in the lower lateral abdomen; however, when the ureter is shortened, it may be difficult to mobilize the ureter to the contralateral stoma site. For such situations, we previously developed a bilateral umbilical tubeless CU using the Toyoda method [2, 4]. The Toyoda method is one of the established techniques for tubeless cutaneous ureterostomy [4] and is routinely used at our institution; therefore, we selected this approach. A distinctive feature of this technique is that the ureter runs laterally and caudally to the lower pole of the kidney within the retroperitoneal space and then courses from dorsal to ventral along the abdominal wall toward the umbilicus. This configuration reduces exposure to the retroperitoneal environment, including potential invasion by metastatic lymph nodes or retroperitoneal metastases. Furthermore, because neither ureteroureteral nor ureteroileal anastomosis is required and both ureters remain separate, direct access to the upper urinary tract is preserved, facilitating selective renal urinary cytology, future BCG instillation therapy, and unilateral endourological surgery. Recent reports have also demonstrated the feasibility of laparoscopic tubeless umbilical CU [5].

The risk of UTUC after RC is higher in patients with CIS or positive ureteral margins [1]. In this case, both risk factors were present, placing the patient at high risk of subsequent upper urinary tract recurrence. Accordingly, we selected this technique because the ureters were shortened after repeated frozen‐section resections and synchronous or metachronous UTUC was suspected.

Laparoscopic RNU after urinary diversion can be technically demanding, and more broadly its surgical setup remains unstandardized [6, 7]. In the present case, we used the same patient positioning and trocar placement routinely employed for laparoscopic left radical nephrectomy at our institution. The ureter coursed ventrally and extraperitoneally toward the umbilicus, allowing relatively straightforward identification of the distal ureter without ureteroileal anastomotic takedown, which is often required in intestinal urinary diversion procedures such as ileal conduit or orthotopic neobladder reconstruction. Reoperation after intestinal urinary diversion can be challenging because of adhesions around the diversion and anastomotic sites [8]. In this case, adhesions between the distal ureter and small intestine required combined small‐bowel resection. However, because umbilical CU does not require intestinal resection or ureteroileal anastomosis, ureteral dissection during reoperation may be technically simplified.

Although the present report describes a single case and this technique cannot replace established urinary diversion methods, it may represent a useful alternative option in selected clinical situations, particularly when future upper urinary tract surgery may be required. Further accumulation of similar cases is warranted to clarify reproducibility and generalizability.

## 4 Conclusion

To the best of our knowledge, this is the first report describing RNU performed after umbilical CU. This urinary diversion may represent a reasonable option for selected patients at high risk of upper urinary tract recurrence.

## Ethics Statement

The authors have nothing to report.

## Consent

Informed consent was obtained from the patient for the publication of this case.

## Conflicts of Interest

The authors declare no conflicts of interest.

## Data Availability

The data that support the findings of this study are available from the corresponding author upon reasonable request.
